# Descriptive review and evaluation of the functioning of the International Health Regulations (IHR) Annex 2

**DOI:** 10.1186/1744-8603-8-1

**Published:** 2012-01-10

**Authors:** Aranka Anema, Eric Druyts, Helge G Hollmeyer, Maxwell C Hardiman, Kumanan Wilson

**Affiliations:** 1 Experimental Medicine Program, Department of Medicine, Faculty of Medicine (2775 Laurel Street), University of British Columbia, Vancouver (V5Z 1M9), Canada; 2 Faculty of Health Sciences (2016-451 Smyth Road), University of Ottawa, Ottawa (K1H 8M5), Canada; 3 Global Capacities Alert and Response, World Health Organization, (20 Avenue Appia, CH 1121) Geneva 27, Switzerland; 4 Department of Medicine, Ottawa Hospital Research Institute, Clinical Epidemiology Program (1053 Carling Avenue), University of Ottawa, Ottawa (K1Y 4E9), Canada

**Keywords:** International Health Regulations (IHR), World Health Organization (WHO), Annex 2, public health emergency of international concern (PHEIC), evaluation

## Abstract

**Background:**

The International Health Regulations (IHRs) (2005) was developed with the aim of governing international responses to public health risks and emergencies. The document requires all 194 World Health Organization (WHO) Member States to detect, assess, notify and report any potential public health emergency of international concern (PHEIC) under specific timelines. Annex 2 of the IHR outlines decision-making criteria for State-appointed National Focal Points (NFP) to report potential PHEICs to the WHO, and is a critical component to the effective functioning of the IHRs.

**Methods:**

The aim of the study was to review and evaluate the functioning of Annex 2 across WHO-reporting States Parties. Specific objectives were to ascertain NFP awareness and knowledge of Annex 2, practical use of the tool, activities taken to implement it, its perceived usefulness and user-friendliness. Qualitative telephone interviews, followed by a quantitative online survey, were administered to NFPs between October, 2009 and February, 2010.

**Results:**

A total of 29 and 133 NFPs participated in the qualitative and quantitative studies, respectively. Qualitative interviews found most NFPs had a strong working knowledge of Annex 2; perceived the tool to be relevant and useful for guiding decisions; and had institutionalized management, legislation and communication systems to support it. NFPs also perceived Annex 2 as human and disease-centric, and emphasized its reduced applicability to potential PHEICs involving bioterrorist attacks, infectious diseases among animals, radio-nuclear and chemical spills, and water- or food-borne contamination. Among quantitative survey respondents, 88% reported having excellent/good knowledge of Annex 2; 77% reported always/usually using Annex 2 for assessing potential PHEICs; 76% indicated their country had some legal, regulatory or administrative provisions for using Annex 2; 95% indicated Annex 2 was always/usually useful for facilitating decisions regarding notifiability of potential PHEICs.

**Conclusion:**

This evaluation, including a large sample of WHO-reporting States Parties, found that the IHR's Annex 2 is perceived as useful for guiding decisions about notifiability of potential PHEICs. There is scope for the WHO to expand training and guidance on application of the IHR's Annex 2 to specific contexts. Continued monitoring and evaluation of the functioning of the IHR is imperative to promoting global health security.

## Introduction

In 2005 the World Health Assembly approved revisions to the International Health Regulations(IHR), the primary document governing the international response to public health risks and emergencies [1,2]. The IHR(2005), which came into force on June 15 2007, require all States Parties (i.e. countries that have ratified a covenant or a convention and are thereby bound to conform to its provisions) to develop and maintain effective national capacities to detect, assess, notify and report events and to respond to public health risks and emergencies [1]. The IHR(2005) represent an important step in achieving global health security by promoting the preparation for, and response to, public health risks and emergencies in a manner that does not unnecessarily impact cross-border travel and trade [3,4].

A major innovation of the IHR(2005) was the shift away from disease specific notification to require notification of any events that may constitute a potential "public health emergency of international concern" (PHEIC) [5]. Under the IHR(2005), PHEICs are not limited to infectious diseases, but also apply to events stemming from biological, radionuclear or chemical agents, from newly discovered or unknown agents or modes of transmission, and events transmissible via persons, vectors, cargo, goods and environmental diffusion [6]. While the first and only deceleration of a PHEIC by the Director General of the World Health Organization (WHO) was the H1N1 outbreak, events such as the export of melamine contaminated foods detected in 2008, the international spread of measles through travelers, the meltdown of a Japanese nuclear power plant in 2011 and the recent E.Coli outbreak in Europe have all been considered potential PHEICs [7,8].

In order to assist States Parties in determining whether a potential PHEIC should be reported to the WHO, the IHR(2005) requires all States Parties to carry out an assessment of public health events arising in their territories using a decision instrument contained in Annex 2 of the Regulations. Under Annex 2, notification by States Parties to WHO must occur if the response to two of four criteria is affirmative, or if an event constitutes any of the following: poliomyelitis, smallpox, human influenza caused by a new subtype, Severe Acute Respiratory Syndrome (SARS), cholera, plague, yellow fever, viral hemorrhagic fevers, West Nile virus, or diseases of regional concern such as meningococcal disease and dengue) (see Additional File 1). States Parties are required to notify the WHO of all qualifying events within 24 hours of confirmation [6].

Annex 2 is considered a critical component to the effective functioning of the Regulations, since its goal is to expand the number and scope of events reported to the WHO by States Parties, thereby strengthening WHO's capacity to monitor and pro-actively respond to public health risks and emergencies. The World Health Assembly (WHA) mandated the Director-General of WHO to review and evaluate the functioning of decision-making tool [1]. The University of Ottawa was commissioned by the WHO to undertake a qualitative study and quantitative survey to explore States Parties awareness, practical use of, usefulness and perceived user-friendliness of Annex 2 through an interview among a representative sample of States Parties (qualitative study) and an online survey involving all States Parties (quantitative study).

## Methods

This review and evaluation of the functioning of Annex 2 was conducted between October 2009 and February 2010, and consisted of two consecutive studies: first, a qualitative study based on semi-structured telephone interviews, and second, a quantitative online survey. In both studies the objectives were to assess the following among State Parties: a) awareness of Annex 2; b) comprehension of the purpose and content of Annex 2; c) use of Annex 2; d) practical implementation of Annex 2; e) usefulness of Annex 2; f) perceived user-friendliness of Annex 2; g) and challenges and success of Annex 2. Qualitative interviews were used to identify salient themes, and to fine-tune wording of questions, for the larger quantitative survey.

### Study Participants

Study participants for both the qualitative and quantitative studies consisted of National IHR Focal Points (NFPs) from WHO-reporting countries. The term "NFP" denotes an institution or office, rather than individual, that has been designated by its States Party as the WHO-communication centre for potential PHEICs under Article 4 of the IHR [9].

WHO State Parties are responsible for defining the NFPs specific position and role within their existing structures. As a consequence, NFPs vary across Member States in terms of their professional qualifications, institutional locations (ie. government departments), and decision-making abilities. The WHO's National IHR Focal Point Guide describes NFP roles, functions and operational requirements under the IHR [9]. According to the guide, the NFP must be available and accessible at all times (7/24/365) for urgent reciprocal communication (via email, telephone and/or fax) with WHO IHR Contact Points. The NFP is responsible for consolidating national public health event surveillance data from all relevant sectors of government, for communicating with WHO IHR Contact Points, on behalf of the State Party concerned and specifically notifying about potential PHEICs [9].

### Qualitative Study

A total of 29 States Parties were purposively selected for participation in the qualitative study and represented a diversity of geographic regions, size, population demographics, economic development, and epidemiological profiles. The sample was weighted to low- and middle-income States Parties to ensure that their unique perspectives were adequately captured. NFPs from these selected States Parties were invited to voluntarily participate in a 1.5 hour interview by email, and offered interpreters to conduct the interview in the language of their choice. The study team, with input from the IHR Coordination Department of WHO, developed an interview protocol focused on the study objectives and consisting of open-ended questions. During interviews, the study team tested the reliability of NFP responses by means of triangulation, a process which cross-examines emerging themes and considers them valid only once two of three questions produce similar answers. Oral and signed consent were obtained from each participant. Telephone interviews were digitally audio-recorded, and transcribed verbatim [10]. Data analysis was facilitated with the use of a qualitative research software, NVivo (version 8.0) (QSR International Pty Ltd., 2008, Melbourne, Australia) to code and sort the collected qualitative data. Two researchers analyzed data in duplicate to ensure that participants' viewpoints were adequately interpreted [11].

### Quantitative Study

Subsequent to the qualitative interviews, all 193 States Parties were invited to participate in the quantitative survey, with the goal of obtaining an exhaustive sample. Contact details were supplied by the IHR Coordination Department. Participants were invited to voluntarily participate in the study via email. The survey containing an embedded participant consent form, was administered to States Parties via a secure internet-based web portal http://www.QuestionPro.com, and was available in all six official languages of the World Health Assembly. Only one answer was allowed for each State Party. Key variables were measured through dichotomous responses and Likert-type scales. PASW Statistics (version 18.0) (SPSS, Inc., 2009, Chicago, USA) was used to clean the dataset and to generate descriptive statistics. Participation in both studies was on a voluntary basis.

### Ethics

The qualitative and quantitative study methodology underwent ethical review at the University of Ottawa and was exempted from review by the WHO Research Ethics Review Committee.

## Results

### Response Rate

**Figure 1:**
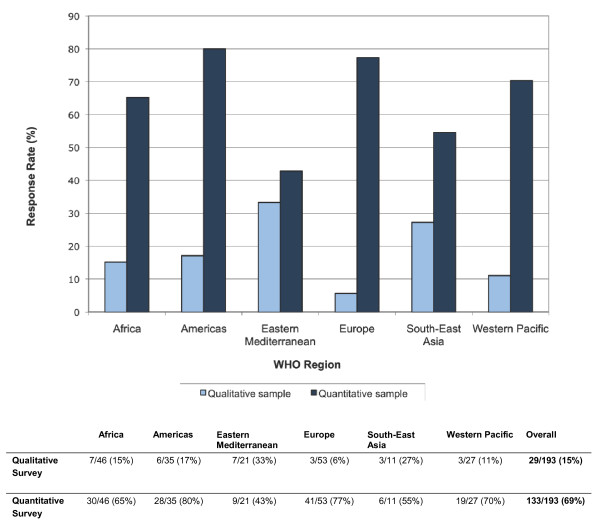
**Participant response rate for qualitative and quantitative surveys, disaggregated by World Health Organization (WHO) region**.

Among the 193 States Parties eligible to participate, a total of 29(15.0%) NFPs participated in qualitative telephone interviews, and 133(68.9%) completed the quantitative online survey. Response rates for the studies varied by WHO region (Figure 1).

### Country Surveillance Capacity

In the quantitative survey, the majority of NFPs reported having either excellent (30[22.9%]) or good (58[44.2%]) ability to assess potential PHEICs under the IHR's Annex 2. NFPs indicated that they received surveillance data from government agencies, to which they apply Annex 2, in the following proportions: Health 127(95.5%), Agriculture 77(57.9%); Environment 45(33.8%); National Security 22(16.5%); Transportation 17(12.8%); and Energy 8(6.0%). NFPs' access to surveillance information was reportedly higher for events involving infectious diseases (Table 1).

**Table 1:** National IHR Focal Point (NFP) reported access to specific types of public health event data for assessment under the International Health Regulations (IHR)(2005)

Types of public health event data	Number (%) NFPs with access to specific types of public health event data (n = 133)
Human influenza caused by new sub-type	126 (94.7%)

Wild-type poliomyelitis	98 (73.7%)

Severe Acute Respiratory Syndrome	79 (59.4%)

Smallpox	60 (45.1%)

Other communicable diseases	94 (70.7%)

Contaminated food (i.e. substance and microbial contamination)	93 (69.9%)

Contaminated water	76 (57.1%)

Radionuclear spill	38 (28.6%)

Chemical contamination of products or the environment	52 (39.1%)

Other toxic release	33 (24.8%)

Bioterrorist attack	45 (33.8%)

Pharmaceutical product (contamination, adverse event, failure)	64 (48.1%)

Communicable diseases among animals	81 (60.9%)

### Awareness and knowledge of Annex 2

NFPs participating in qualitative interviews were unanimously aware of Annex 2, and had varying in-depth knowledge about the tool, depending on their prior exposure to WHO guidance and trainings. Some confusion persisted among NFPs that had not accessed any training regarding operational and communication procedures for notifying WHO about potential PHEICs. In the quantitative survey, 112(88.2%) NFPs indicated they had 'excellent' or 'good' knowledge of Annex 2, and 108(82.1%) had accessed some form of WHO training about Annex 2. When we explored the statistical association between NFPs knowledge of Annex 2 and access to training, we found that NFPs who had accessed WHO training were significantly more likely to report having 'excellent' or 'good' knowledge of Annex 2, compared to those that had not accessed any training (p = 0.03).

Qualitative interviews with NFPs also revealed that many States Parties were expanding awareness and use of Annex 2 beyond the national/federal level, to state and municipal public health officials and even front-line clinicians. In the quantitative survey, 37(29.8%) NFPs rated the overall awareness of Annex 2 at the national/federal government level as 'excellent' or 'good', compared to only 19(16.1%) at the provincial/state/canton and 13(10.1%) at municipal/local levels. A total of 93(70.5%) of NFPs reported 'excellent' or 'good' awareness of Annex 2 in Health agencies, compared to 32(24.8%) in Agriculture, 21(16.4%) in Environment, 20(15.6%) in National Security, 13(10.2%) in Transportation, 12(9.4%) in Justice, and 7(5.6%) in Energy.

### Practical Use of Annex 2

#### Frequency of Use

The qualitative interviews revealed that NFPs are using Annex 2 with varying levels of frequency. While some have instituted a routine practice of applying Annex 2 to all public health events that emerge in their national surveillance system, others reserve the tool for events they suspect may qualify for notification under Annex 2's criteria. The quantitative survey revealed that 59(46.8%) respondents indicating they 'always' use Annex 2 and 38(30.2%) indicating they 'usually' use the tool for the notification assessment of potential PHEICs.

#### Training

The qualitative interviews revealed that many NFPs are leading trainings about Annex 2 within their countries through a range of mechanisms. Some NFPs indicated they had conducted informal trainings about Annex 2, while others had developed elaborate training curricula and 'trainers of trainers' for the purpose of increasing knowledge, institutional memory and succession planning regarding Annex 2. In the quantitative survey, 84(67.2%) NFPs indicated they had facilitated trainings about Annex 2, which were conducted largely within government health agencies (Table 2).

**Table 2:** Number of National IHR Focal Points (NFPs) that reported facilitating trainings about Annex 2 of International Health Regulations (IHR) in specific government agencies

Type of government agency	Number (%) trainings facilitated by NFPs (n = 133)
Health	83 (62.4%)

Agriculture	49 (36.8%)

National Security	33 (24.8%)

Environment	33 (24.8%)

Transportation	30 (22.6%)

Justice	10 (7.5%)

Energy	7 (5.3%)

#### Legal, Regulatory or Administrative Instruments

During the qualitative interviews, many NFPs explained that their country had some form of legislation pertaining to Annex 2 in place, usually regarding infectious diseases. In the quantitative survey, 94(76.4%) NFPs responded that their country had some form of legal, regulatory or administrative provisions for assessing and notifying public health events in accordance with Annex 2, either in place or under development. Of these States Parties, 33(35.9%) had guidelines to facilitate interpretation of this federal/national legislation regarding Annex 2. Consistent with qualitative findings, 91(68.4%) NFPs indicated they had legislation concerning the four diseases requiring notification in all circumstances under the IHR (2005)'s Annex 2: 91(68.4%) human influenza caused by a new subtype; 82(61.7%) for wild-type poliomyelitis; 85(63.9%) for SARS; and 75(56.4%) for smallpox.

#### Standard Operating Procedures (SOPs)

Qualitative interviews with NFPs revealed that several States Parties had Standard Operating Procedures (SOPs) that provided guidance on the deployment of human/financial resources, inter/intra-agency and public communication, and other operation-based activities pertaining to the assessment and notification of potential PHEICs under Annex 2. Several countries indicated they had SOPs for specific locations within their territory and for certain scenarios. In the quantitative survey, 68(54.4%) NFPs indicated that they had a formal SOP for the implementation of Annex 2 either in place or in development. Among these State Parties 39(29.2%) had SOPs to guide assessment and notification of potential PHEICS for points of entry, 28(21.1%) for ships, 28(21.1%) for front-line public health settings (e.g. clinics, hospitals), 16(12.0%) for animal farms, 11(8.3%) for food processing plants, 9(6.8%) for water treatment plants, 8(6.0%) for international population gatherings, and 7(5.3%) for industrial plants.

#### Communication Systems

During the qualitative interviews, several NFPs explained they had domestic communication plans in place to ensure timely detection, assessment and monitoring of potential PHEICs within their country. Many NFPs expressed a desire to improve their communication with international stakeholders including WHO, but had varying levels of comfort regarding early communication about surveillance data with WHO officials and NFPs from neighboring countries. Predominant concerns regarding early communication with WHO officials and NFPs from neighboring countries included the fear that it may raise unnecessary alarm in WHO and neighboring governments, may cause excessive media attention and may lead to unnecessary travel or trade restrictions (Table 3). In the quantitative survey, 99(74.4%) of NFPs responded that they had a domestic communications plan in place or in development to facilitate intra-country communication about public health events of potential international concern.

**Table 3:** National IHR Focal Points (NFPs) concerns about early communication with World Health Organization (WHO) and with NFPs from neighboring countries regarding a potential public health emergency of international concern (PHEIC)

NFP concerns regarding early communication	Communication with WHO country and regional offices (n = 133)	Communication with NFPs from neighboring States Parties (n = 133)
	
	Yes (%)	Yes (%)
**Early communication may unnecessarily raise alarm in WHO**	31 (23.3%)	15 (11.3%)

**Early communication may unnecessarily raise alarm in my government**	33 (24.8%)	20 (15.0%)

**Early communication may unnecessarily raise alarm in the government of a neighboring country**	21 (15.8%)	41 (30.8%)

**Early communication may create unnecessary media attention**	37 (27.8%)	33 (24.8%)

**Early communication may result in unnecessary trade/travel restrictions**	24 (18.0%)	29 (21.8%)

**Early communication may overburden country and regional offices and NFPs**	14 (10.5%)	15 (11.3%)

**Early communication utilizes our limited telephone communications budget**	6 (4.5%)	4 (3.0%)

**None of the above**	56 (42.1%)	60 (45.1%)

**Other**	8 (6.0%)	6 (4.5%)

### Usefulness of Annex 2

In the qualitative interviews, most NFPs expressed that they found Annex 2 to be very useful for deciding upon the need to notify WHO of a potential PHEIC, particularly for those PHEICs deemed automatically notifiable under Annex 2 (see Additional File 1). However, some raised concerns that, in the absence of any accepted, evidence-based hierarchy of pathogens and toxins, Annex 2 was difficult to apply to certain events such as human influenza caused by a new subtype, food- and water-borne events, and chemical spills. Several NFPs described Annex 2 as overly human-centric, limiting its application to potential PHEICs consisting of communicable diseases among animals. In the quantitative survey, 116(95%) NFPs indicated that Annex 2 was 'always' or 'usually' useful in facilitating decisions regarding whether a public health event has to be notified to WHO. NFPs found Annex 2 'fully relevant' for infectious diseases, such as smallpox (114[87.7%]), SARS (110[84.0%]), and human influenza cause by a new subtype (110[84.0%]), and less relevant for communicable diseases among animals 35[27.1%]), chemical contamination of products or the environment (57[44.5%]), and contaminated water (52[40.6%]) or foods (5[41.4%]).

### User-friendliness of Annex 2

During the qualitative interviews most NFPs indicated that the 24-hour timeline for notification of a potential PHEIC to WHO was reasonable, but delays were inevitable due to the need to obtain clearance from senior government officials. In the survey, 113(89.0%) NFPs reported that the 24-hour timeline for notification was reasonable. However, 51(40.2%) NFPs require clearance from 2-3 individuals/offices prior to notification to WHO, contributing to delays in notification.

Interviews revealed that the majority of NFPs felt that the four decision instrument criteria of Annex 2 were clear but could benefit from refinements in the algorithm and checklist in order to prevent difficulties in interpretation. In the quantitative survey, NFPs indicated that the user-friendliness of Annex 2 could be improved if NFPs had access to disease-specific incidence threshold values to facilitate assessment of each criterion (67[50.4%]), guidance on how to interpret surveillance data in the specific national context (63[47.4%]), and more case scenarios for training (90[67.7%]). Just over half of all NFPs additionally suggested that the development of a centralized online communication platform would be useful in order to expand notification options, improve communication between NFPs from neighboring countries and contribute to training in the use of Annex 2 (68[56.7%]).

## Discussion

Overall, our findings suggest that there is overwhelming support for Annex 2 among NFPs. Although States Parties appear to have varied capacities in event-based surveillance, we found that the vast majority of NFPs had a strong awareness and knowledge of Annex 2, particularly those within government health agencies. Annex 2 was deemed useful for assessing communicable diseases, and less helpful for discerning other types of potential PHEICs. NFPs cited numerous initiatives to support the practical use of the tool and provided several suggestions on how to improve its user-friendliness.

Our results indicate that States Parties' ability to detect potential PHEICs was strongest in government health agencies, and lowest in agencies of national security, transportation and energy. Results from this study suggest that many States Parties may be struggling to establish core capacities in event-based surveillance [12]. The IHR (2005) calls upon State Parties to enhance their surveillance and response infrastructure and necessary logistical and human resource capacity across all governments sectors by 2012 [13]. However, for many low resource countries, the development of an epidemic intelligence framework across multiple sectors, as has been done in the European Union for example [14], poses a serious financial challenge [15] and may explain the significant variation between States Parties' reported surveillance capacity [16-19]. These findings suggest there is scope for the WHO to further support States Parties in enhancing their national surveillance, potentially by leveraging existing bilateral partnerships focused on capacity building [20].

We also found that the majority of NFPs regularly used Annex 2 for the assessment of public health events, and had taken active steps towards institutionalizing its use in their national, regional and municipal surveillance systems. The majority of NFPs had facilitated trainings about Annex 2 in their country, many had developed general SOPs and systems to facilitate rapid communication of public health events from municipal to national levels of government. Of note, the vast majority of States Parties had some form of legal, regulatory or administrative provisions supporting the use of Annex 2 and many had guidelines to facilitate its interpretation. These findings suggest that most States Parties are meeting their IHR core capacity requirements for the establishment of national legislation and policy [12], and that many federations, where public health regulatory power resides in local or regional governments, may be centralizing and harmonizing their public health policies and practices, allowing them to better comply with the IHR(2005) [21-23]. The fact that most States Parties indicated they had legislation specific to the four diseases requiring automatic under the IHRs Annex 2, and had SOPs to guide use of Annex 2 in diverse settings, suggests that cross-national management systems are in place to effectively notify WHO of potential PHEICs.

Our findings also suggest that NFPs generally perceived Annex 2 to be very user-friendly. NFPs cited the timeline for notification of a potential PHEIC as reasonable and that the algorithm and checklists represented a substantial improvement over the previous IHR disease list. Overall, NFPs felt that Annex 2 was simple to read and clear, particularly when applied to communicable diseases. However, NFPs described having difficulties in gauging the severity of certain types of public health events, given the absence of evidence-based thresholds (e.g. contamination of food and water, infectious diseases among animals and chemical contamination of products or the environment). These findings are consistent with a published reported from one State Party in sub-Saharan Africa that indicated the country lacked surveillance guidelines and case definitions for outbreak response to food, chemical and radio-nuclear hazards [18]. In developing countries, effective detection of food and water-borne diseases requires significant improvements in laboratory infrastructure and expertise [24]. Meanwhile, the surveillance and reporting of chemical, nuclear and radiological threats have been described as persistent challenges by several States Parties in both developing and developed countries [25], complicating planning for major incidents [26]. Further guidance where possible, on global standardization of rare types of public health events were deemed necessary by NFPs.

Our findings regarding NFP awareness, knowledge and efforts to integrate Annex 2 into national legislation, organizational procedures and communication systems appears to be in direct contrast with results from the recent WHO Database Study which found that notification of public health events by NFPs has remained quite limited [27]. A potential explanation for this discordance is that Annex 2 was designed and written in such a way as to be intentionally non-specific. It has been assumed that this intentional ambiguity would broaden the type and numbers of notifiable events under the IHR (2005) and lead to an over-reporting of public health events by National IHR Focal Points to WHO. However, that intentional ambiguity may actually be having the opposite effect. Lack of detail in Annex 2 may in fact have allowed more discretion in reporting which in turn could have resulted in more conservative notification practices.

### Implications of Findings

There are several steps WHO and States Parties can take to further improve the use of Annex 2 (Additional File 2). Since having a thorough and confident understanding of Annex 2 was associated with having accessed WHO guidance and training on the tool, there is a need to ensure that all NFPs access some form of training regarding Annex 2, and especially the WHO's Guidance for the Use of Annex 2 of the IHR(2005) [28]. A mechanism to prevent the non-specificity of Annex 2 as a reason to err on the side of not reporting and to support NFPs in any internal disputes over notification, would be to provide more specific examples of what classes of conditions would require reporting through an increased number of case scenarios. If a case study of an analogous event suggested that reporting is required it could reduce discretion resulting in the decision to not report. NFPs unanimously found the case scenarios contained within WHO's Interim Guidance for the Use of Annex 2 of the IHR(2005)[6] to be helpful for obtaining a strong working knowledge of the tool.

Several States Parties have demonstrated a tremendous amount of innovation with regard to activities they have taken to support Annex 2. In some circumstances, these activities to support Annex 2 may constitute 'best practices' that other States Parties can learn from and warrant closer attention. There is equally a need for WHO to develop parameters for the appropriation/modification of Annex 2 by States Parties. Furthermore, while the intention and one of the great strengths of Annex 2 is to require an interpretation of public health events taking into account the context in which they occur, there is scope for WHO to support NFPs in their notification assessment by developing thresholds for the seriousness and risk of spread for specific events and circumstances.

The majority of NFPs supported the use of a centralized, web-based platform to simultaneously strengthen training in the use of Annex 2, information sharing with NFPs from neighboring States Parties, and notification of potential PHEICs to WHO, Internet-based reporting has been associated with increased timeliness of outbreak detection and public communication [29], and is becoming increasingly feasible in developing countries due to growing Internet access, IT user-friendliness and reduced costs [30]. Automated syndromic surveillance system could complement existing laboratory and public health surveillance programs, and be maintained with minimal investment into technological or human resources [30].

### Study Limitations

When considering the findings of our study it is important to recognize the limitations of the methodology. First, our evaluation sampled NFPs. These individuals would be expected to be amongst the most supportive and knowledgeable individuals of the IHR (2005) within a State Party. Similar enthusiasm and knowledge for the IHR cannot necessarily be expected to exist in other parts of the public health surveillance and response system and could reflect on the ability of a States Party to utilize Annex 2. Furthermore NFPs may not necessarily play the key role in the risk assessment of an event occurring within the territory of a given State Party. This process may involve decision makers based outside the respective NFP. While the studies were addressed to NFPs, the only national stakeholder clearly identifiable and accessible by WHO, no restrictions were imposed on NFPs regarding consultation with other relevant decision makers. However, because of the anonymous nature of the survey we do not know whether the answers that we received from NFPs represent the views of individual risk assessors within the NFP, the entire NFP team, or a group of collaborators including national experts outside of the NFP. Additionally, our findings are susceptible to responder bias. NFPs that did participate may have been systematically different from those that did not. In particular we noted a differential response per WHO Region, with comparatively less responses from the Eastern Mediterranean and South East Asia, and more responses from the Americas and Europe. Non-response from certain States Parties may be explained by individual circumstances (e.g. workload), cultural norms, or participant exhaustion (from other recent WHO evaluation [31]). Future evaluations should seek to verify whether observations from the present study are representative of those regions. Also, for those NFPs we obtained responses from, there is the risk of social desirability bias. It is possible, for example, that participants modified their responses (e.g. regarding awareness, knowledge, usefulness of Annex 2) in order to satisfy WHO headquarters representatives associated with the study. Finally, descriptive results from this study should be interpreted as baseline data for subsequent in depth analysis and longitudinal investigation.

## Conclusion

Our findings suggest that there is overwhelming support for Annex 2 among States Parties. Many States Parties had taken active steps towards institutionalizing the IHR's Annex 2 in their national, regional and municipal surveillance systems, suggesting State commitment to the development of IHR core capacities. States Parties' ability to detect potential PHEICs was strongest in government health agencies, and lowest in agencies of national security, transportation and energy, pointing towards areas for possible expansion of WHO-supported capacity building efforts. The IHR's Annex 2 was deemed highly useful for assessing notification of infectious diseases, but less helpful for evaluating other types potential PHEICs, suggesting scope for the WHO to expand and refine its guidance documents.

## List of abbreviations

IHR: International Health Regulations; NEP: National IHR Focal Point; PHEIC: public health emergency of international concern; WHO: World Health Organization.

## Competing interests

The authors declare that they have no competing interests.

## Authors' contributions

AA and KW led and implemented the independent program evaluation, which involved developing qualitative and quantitative study designs and survey tools; leading participant interviews; analyzing study results. AA and KW wrote the manuscript. ED assisted data collection, cleaning and presentation of results. MH and HH advised study design and contributed to discussion of results. All authors read and approved the final manuscript.

## Supplementary Material

Additional file 1 **Annex 2 of the International Health Regulations (IHRs): Decision instrument for the assessment and notification of events that may constitute a public health emergency of international concern**. Jpeg figure. Click here for file

Additional file 2 **World Health Organizations (WHO) recommendations to strengthen the functioning the International Health Regulations (IHR) Annex 2**. Recommendations listed from 1 to 15. Click here for file
