# Supplementary figures and images for: Descriptive review and evaluation of the functioning of the International Health Regulations (IHR) Annex 2
Source: Global Health. 2012 Jan 10;8:1. doi: 10.1186/1744-8603-8-1 (PMC3313850; doi:10.1186/1744-8603-8-1)

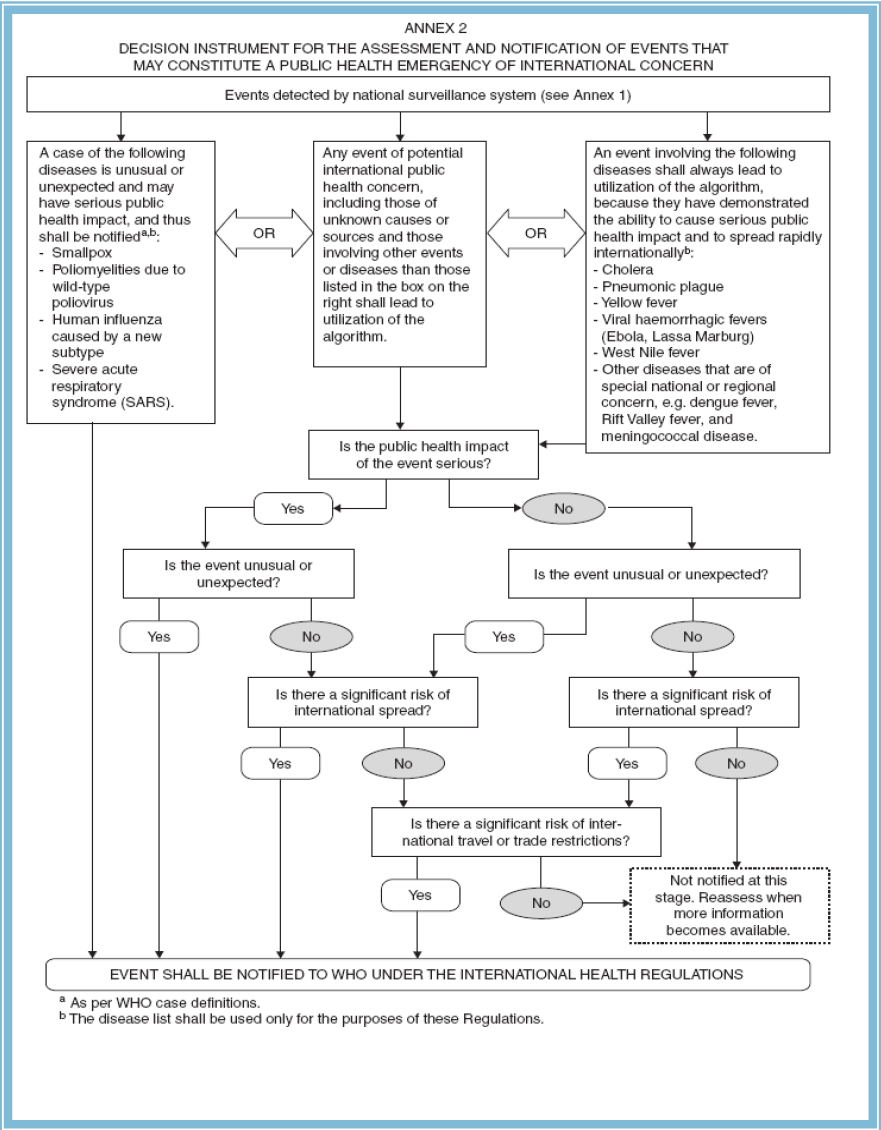

Supplement: Additional file 1 — Annex 2 of the International Health Regulations (IHRs): Decision instrument for the assessment and notification of events that may constitute a public health emergency of international concern. Jpeg figure. [file 1744-8603-8-1-S1.DOC]
