# Supplementary material for: Descriptive review and evaluation of the functioning of the International Health Regulations (IHR) Annex 2
Source: Global Health. 2012 Jan 10;8:1. doi: 10.1186/1744-8603-8-1 (PMC3313850; doi:10.1186/1744-8603-8-1)
Supplement: Additional file 2 — World Health Organizations (WHO) recommendations to strengthen the functioning the International Health Regulations (IHR) Annex 2. Recommendations listed from 1 to 15. [file 1744-8603-8-1-S2.DOC]

**WORLD HEALTH ORGANIZATION (WHO) RECOMMENDATIONS TO STRENGTHEN THE FUNCTIONING OF THE INTERNATIONAL HEALTH REGULATIONS (IHR) ANNEX 2**

*Expand and Standardize Knowledge about Annex 2*

1. Encourage all National IHR Focal Points (NFPs) to review the WHO’s Interim Guidance for the Use of Annex 2 of the IHR (2005) within a specific time-frame of taking their position;
2. Support NFPs in the expansion of knowledge/awareness about Annex 2 to non-health sectors of government, and notably to agencies of Energy and Justice;
3. Support NFPs in sensitization of government agencies about Annex 2 through development of political advocacy tools for high-level officials.

*Strengthen and Standardize Practical Use of Annex 2*

1. Consider promoting sharing of intra-country training modules and ‘trainer of trainers’ curricula that have been developed by States Parties to support their use of Annex 2;
2. Strengthen support to States Parties for the development of legislation specific to non-infectious disease events; consider guiding States Parties in their development of inter-sectoral legislation pertaining to Annex 2 at the municipal/local level;
3. Consider developing general SOPs for the implementation of Annex 2, based on a review of international best practices and lessons learned;
4. Facilitate inter-country sharing of SOPs pertaining to specific types of public health events of potential international concern;
5. Place additional emphasis on Article 4 to ensure that NFPs understand their responsibility to notify their IHR Contact Point at the Regional Office or at Headquarters (as appropriate);
6. Further promote Article 8 to NFPs, encouraging informal telephone and other communication with IHR Contact Points regarding public health events under assessment;
7. Promote Article 44 and encourage greater collaboration/communication between NFPs from neighboring States Parties for the purpose of detection, assessment and response to events notifiable under the IHR;

*Improve User-friendliness of Annex 2*

1. Consider expanding the number and breadth of case scenarios in WHO’s Interim Guidance for the Use of Annex 2 of the IHR (2005) for the purpose of training and guiding NFPs; include additional scenarios regarding radio-nuclear and chemical events, bio-terrorist attacks, food and water contamination, and communicable diseases among animals.
2. Provide NFPs with further guidance on how to interpret public health event of potential international concern in light of the compounding effects of: i) proximity to international border/points of entry; ii) national/local capacity response capacity; iii) projected economic impact; iv) additional risk of accidents; v) need for immediate international response; vi) impact on international human security; vii) time of the outbreak; and viii) presence of animal reservoirs that may affect humans.
3. Develop parameters for disallowable and allowable modifications to Annex 2’s algorithm and checklist;
4. Consider providing NFPs with more detailed epidemiological guidance, specifically on communicable diseases that have been problematic (e.g. food and water borne diseases, communicable diseases among animal) and non-communicable diseases with which NFPs have had limited experience (e.g. radio-nuclear events, chemical spills, bioterrorist attacks);
5. Consider the development of a secured and centralized online platform, containing an interactive version of Annex 2 in order to: i) expand interactive training of NFPs in use of Annex, ii) promote real-time assessment of public health events; iii) expand options for notification to WHO; iv) encourage communication between NFPs from neighboring countries; ad v) stimulate the sharing of documents between NFPs.
